# Kidney Tubular Damage Secondary to Deferasirox: Systematic Literature Review

**DOI:** 10.3390/children8121104

**Published:** 2021-12-01

**Authors:** Martin Scoglio, Maria Domenica Cappellini, Emanuela D’Angelo, Mario G. Bianchetti, Sebastiano A. G. Lava, Carlo Agostoni, Gregorio P. Milani

**Affiliations:** 1 Department of Pediatrics, Pediatric Institute of Southern Switzerland, Ospedale San Giovanni, 6500 Bellinzona, Switzerland; martin.scoglio91@gmail.com (M.S.); mario.bianchetti@usi.ch (M.G.B.); 2 Pediatric Unit, Fondazione IRCCS Ca’ Granda Ospedale Maggiore Policlinico, 20122 Milan, Italy; maria.cappellini@unimi.it (M.D.C.); emanuela.dangelo@policlinico.mi.it (E.D.); carlo.agostoni@unimi.it (C.A.); milani.gregoriop@gmail.com (G.P.M.); 3 Pediatric Cardiology Unit, Department of Pediatrics, Centre Hospitalier Universitaire Vaudois, and University of Lausanne, 1010 Lausanne, Switzerland; 4 Heart Failure and Transplantation, Department of Pediatric Cardiology, Great Ormond Street Hospital, London WC1N 3JH, UK; 5 Department of Clinical Sciences and Community Health, Università degli Studi di Milano, 20122 Milan, Italy

**Keywords:** deferasirox, kidney tubular damage, chelator, iron overload, transfusion

## Abstract

Deferasirox is a first-line therapy for iron overload that can sometimes cause kidney damage. To better define the pattern of tubular damage, a systematic literature review was conducted on the United States National Library of Medicine, Excerpta Medica, and Web of Science databases. Twenty-three reports describing 57 individual cases could be included. The majority (*n* = 35) of the 57 patients were ≤18 years of age and affected by thalassemia (*n* = 46). Abnormal urinary findings were noted in 54, electrolyte or acid–base abnormalities in 46, and acute kidney injury in 9 patients. Latent tubular damage was diagnosed in 11 (19%), overt kidney tubular damage in 37 (65%), and an acute kidney injury in the remaining nine (16%) patients. Out of the 117 acid–base and electrolyte disorders reported in 48 patients, normal-gap metabolic acidosis and hypophosphatemia were the most frequent. Further abnormalities were, in decreasing order of frequency, hypokalemia, hypouricemia, hypocalcemia, and hyponatremia. Out of the 81 abnormal urinary findings, renal glucosuria was the most frequent, followed by tubular proteinuria, total proteinuria, and aminoaciduria. In conclusion, a proximal tubulopathy pattern may be observed on treatment with deferasirox. Since deferasirox-associated kidney damage is dose-dependent, physicians should prescribe the lowest efficacious dose.

## 1. Introduction

Iron overload secondary to regular blood transfusions may result in injury and dysfunction of the heart, liver, anterior pituitary, pancreas, and joints. Both parenteral iron chelation with deferoxamine and oral chelation with deferiprone or deferasirox have been shown to reduce iron overload and organ damage [1,2]. Due to its efficacy and ease of use, deferasirox 20–30 mg/kg once-daily is currently the first-line therapy for iron overload secondary to blood transfusions [1,2]. It has been known for about 10 years that an increase in circulating creatinine occurs in about one out of ten cases [3]. This tendency is most likely to occur in well-chelated patients with circulating ferritin 1000 µg/L or less [4]. The pattern of kidney tubular damage that occurs with deferasirox therapy, however, is poorly defined. To address this issue, we performed a systematic review of the individually reported cases of kidney tubular damage that have been associated with deferasirox.

## 2. Materials and Methods

### 2.1. Search Strategy

This review was accomplished following the Preferred Reporting Items for Systematic Reviews and Meta-Analyses recommendations [5]. Searches without time or language restrictions were run in the databases of the United States National Library of Medicine, Excerpta Medica, and Web of Science in November 2020 and repeated during June 2021. Original papers published after 2008 [6] with no language limits were considered. The search strategy incorporated the terms (acidosis OR electrolyte disturbances OR hypercalcemia OR hyperkalemia OR hypermagnesemia OR hypernatremia OR hyperchloremia OR hypocalcemia OR hypokalemia OR hypomagnesemia OR hyponatremia OR hypochloremia OR hypophosphatemia OR hypouricemia OR kidney damage OR renal damage OR glucosuria OR proteinuria OR aminoaciduria) AND (deferasirox OR Exjade OR Jadenu). References listed within bibliographies of the retrieved records and articles already known to the authors were also considered for inclusion. Furthermore, we contacted the authors of the original reports to obtain missing information.

Two authors independently screened all identified titles and abstracts in a non-blinded fashion. Upon retrieval of candidate reports, full-text publications were evaluated for eligibility. Any discordance was resolved by consensus.

### 2.2. Eligibility Criteria

We included individuals on deferasirox therapy found with at least one of the following three laboratory findings: (1) urinary abnormalities such as renal glucosuria (i.e., normoglycemic), increased urinary total protein excretion, excessive tubular proteinuria (ß_2_-microglobulin, retinol-binding protein or N-acetyl-ß-glucosaminidase) or generalized aminoaciduria; (2) otherwise unexplained metabolic acid–base abnormalities (acidosis: bicarbonate ≤18 mmol/L and pH ≤7.38; alkalosis: bicarbonate ≥27 mmol/L and pH ≥ 7.42) or electrolyte disturbances (total calcium ≥2.80 mmol; ionized calcium ≥1.40 mmol/L; total calcium ≤2.20 mmol/L; ionized calcium ≤1.10 mmol/L; total magnesium ≥1.20 mmol/L; total magnesium ≤0.70 mmol/L; sodium ≥146 mmol/L; sodium ≤134 mmol/L; potassium ≥5.1 mmol/L; potassium ≤3.4 mmol/L). Since children and adults importantly differ with respect to uric acid metabolism, age- and gender-dependent reference values were used for the definition of hyper- and hypouricemia [7]; (3) laboratory features consistent [8] with an acute kidney injury (defined as a rise in circulating creatinine level to ≥1.5 times baseline or increase by ≥27 µmol/L).

Patients with pre-existing conditions other than the blood transfusion-dependent disease, patients on other drugs with a potential to induce acid–base or electrolyte disturbances, patients affected by intercurrent infectious disease, and pregnant women were excluded.

### 2.3. Data Extraction—Classification

Data were extracted using a piloted form and transcribed into a predefined spreadsheet. From each case meeting the mentioned inclusion criteria demographics, the underlying transfusion-dependent disease, information on deferasirox therapy before the onset of kidney damage, laboratory values (including urinary findings, acid–base balance, electrolytes, and blood creatinine values), and time to resolution after withdrawing deferasirox were extracted. The possible occurrence of kidney damage after reintroducing deferasirox was also addressed. Finally, since metabolic acidosis is categorized as anion gap and non-anion gap acidosis, this information was collected in patients with this acid–base abnormality. If needed, attempts were made to contact original authors to obtain missing data. The clinical features associated with the kidney damage were poorly documented and were consequently not extracted.

Circulating creatinine was used to classify acute kidney injury [8] as stage I (rise in creatinine by ≤1.9 times baseline), stage II (rise in creatinine by 2.0–2.9 times baseline), and stage III (rise in creatinine by ≥3.0 times baseline, rise in creatinine by ≥354 µmol/L, or the start of kidney replacement therapy).

### 2.4. Completeness of Reporting

Completeness of included cases was judged using the following two components: 1. description of diagnosis and drug treatment (including dose), duration of therapy, and time to recovery of kidney damage (rating 0 to 4); and 2. description of laboratory data in blood and urine (rating 0 to 4). The reporting completeness was graded according to the sum of each item as excellent (≥6), good (4–5), satisfactory (2–4), or poor (<2).

### 2.5. Analysis—Statistics

The pairwise deletion was used to handle missing data. Categorical data are given as frequency and were analyzed using the Fisher’s exact test. Continuous data are given as a median and interquartile range and were analyzed using the Kruskal–Wallis test and the Dunn posttest. Significance was set at *p* < 0.05.

## 3. Results

### 3.1. Search Results—Completeness of Reporting

**Figure 1:**
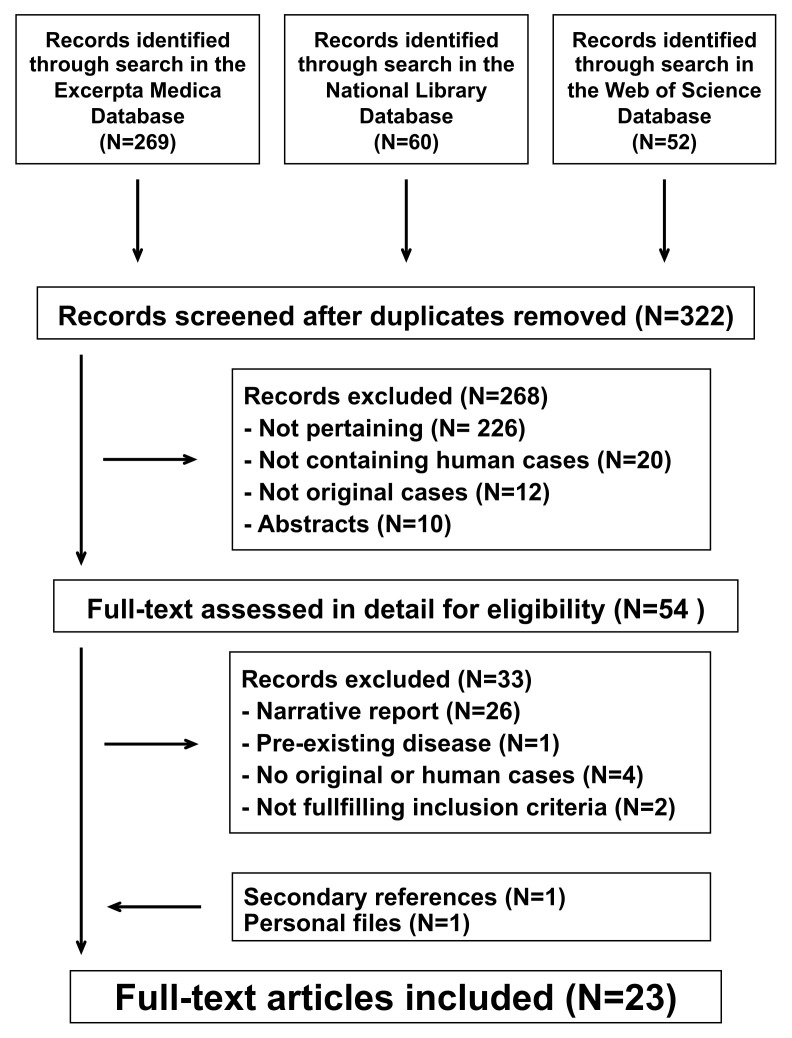
Kidney damage associated with deferasirox therapy. Flowchart of the literature search process.

The literature search process is summarized in Figure 1. For the final analysis, we retained 23 reports [4,6,9,10,11,12,13,14,15,16,17,18,19,20,21,22,23,24,25,26,27,28,29] published between 2009 and 2021 in English. They had been reported from the following countries: United States of America (*N* = 6), Italy (*N* = 3), France (*N* = 3), Australia (*N* = 2), Israel (*N* = 2), Switzerland (*N* = 2), Taiwan (*N* = 2), Canada (*N* = 1), China (*N* = 1), and Greece (*N* = 1). The mentioned articles described 57 individual cases. Reporting completeness was excellent in 9 (16%), good in 21 (37%), and satisfactory in the remaining 27 (47%) cases.

### 3.2. Findings

The majority (61%) of the 57 patients were ≤18 years of age (Table 1). Three-quarters of the cases were affected by a thalassemia syndrome. Laboratory features consistent with kidney damage were mostly observed >6 months after starting a standard dose deferasirox therapy, although this information was not available in more than half of the cases. A recurrence of the kidney damage was noted in nine of the 18 patients, who were again exposed to deferasirox (usually in a reduced dose).

Abnormal urinary findings were noted in 54 (95%), electrolyte or acid–base abnormalities in 46 (81%), and an acute kidney injury in 9 (16%) cases (Table 2). Abnormal urinary findings without any electrolyte or acid–base abnormality and without acute kidney injury were noted in 11 (19%) cases. The diagnosis of latent tubular damage was made in these cases. Nine (16%) cases presented with abnormal urinary findings, electrolyte or acid–base abnormalities, and acute kidney injury. The diagnosis of acute kidney injury accompanied by tubular damage was made in these cases. The remaining 37 (65%) cases presented with abnormalities in the electrolyte or acid–base balance but without any kidney injury (abnormal urinary findings were also observed in 34 out of the 37 cases). Hence, the diagnosis of overt kidney tubular damage was made in these cases. All patients with acute kidney injury (20 [19–33] years) were male. Furthermore, they were slightly but not significantly older than patients with latent (14 [11–19] years) or overt (11 [5.6–20] years) tubulopathy.

A total of 117 acid–base and electrolyte disorders were reported in the 48 patients with either isolated overt tubular injury or acute kidney injury (Table 2). Metabolic acidosis (with a normal anion gap) and hypophosphatemia were reported in more than three-quarters of cases. Further disorders were, in decreasing order of frequency, hypokalemia, hypouricemia, hypocalcemia, and hyponatremia. Metabolic alkalosis, hyperkalemia, hyperuricemia, hypercalcemia, hypernatremia, hypermagnesemia or hypomagnesemia were never identified. 

A total of 81 abnormal urinary findings were detected. Renal glucosuria was the most frequently detected urinary abnormality, followed by tubular proteinuria, total proteinuria, and generalized aminoaciduria.

## 4. Discussion

The present systematic review points out that the kidney tubular damage associated with oral deferasirox therapy may present in three ways: (a) abnormal urinary findings consistent with latent tubular damage; (b) overt acid–base or electrolyte abnormalities; and (c) acute kidney injury (always associated with abnormal urinary findings and with an electrolyte or acid–base imbalance).

The kidney tubular damage caused by deferasirox characteristically presents with renal glucosuria, excessive ß_2_-microglobulin excretion, generalized aminoaciduria, non-gap metabolic acidosis, hypophosphatemia, and hypouricemia. Hence, these data suggest the existence of a proximal tubular disturbance. Proximal tubular defects are typically observed on treatment with aminoglycosides, nucleotide reverse transcriptase inhibitors, or platinum compounds and result from mitochondrial toxicity [30,31]. No kidney damage was so far reported on treatment with other iron chelators such as deferiprone or deferoxamine [2,32]. These clinical observations are supported by in vitro studies: it was found that deferasirox, but not other chelators, induces a dramatic swelling of mitochondria and decreases the cellular ATP content [33]. Iron being essential for kidney tubular cells, it is currently assumed that deferasirox associated tubular damage results from excessive chelation of iron within these cells, which is likely superior to that observed with other chelators [34]. Furthermore, the kidney damage caused by deferasirox is likely dose-dependent [34]. Finally, pharmacogenetics might be a relevant determinant of deferasirox toxicity [34,35].

**Figure 2:**
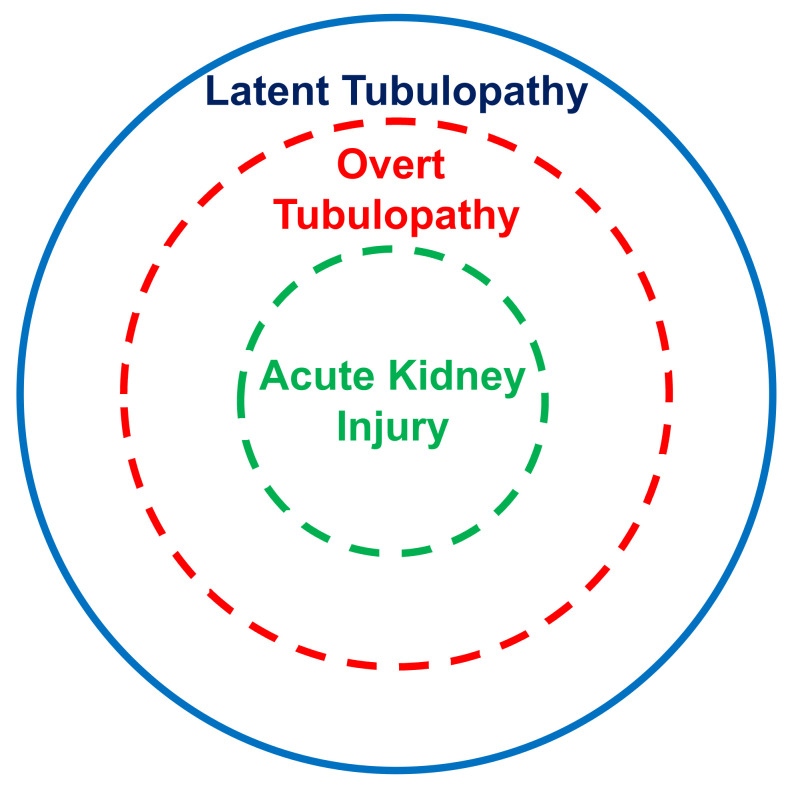
Suggested stratification of the kidney damage associated with deferasirox therapy.

In this analysis, most cases with acid–base or electrolyte abnormalities simultaneously presented with urinary abnormalities. Furthermore, all cases with kidney injury concurrently presented with an acid–base or electrolyte imbalance and with urinary abnormalities. It is therefore tempting to assume that the latent tubulopathy represents the early stage, the overt tubulopathy the middle stage, and finally the manifest acute kidney injury the advanced stage of damage induced by deferasirox (Figure 2). This hypothesis deserves confirmation in longitudinal studies. 

It has been speculated but not proven that iron overload may per se induce kidney damage [1,2]. Furthermore, sickle cell disease and perhaps also some other transfusion-dependent conditions may cause kidney abnormalities [36]. In the present analysis, however, patients affected by sickle cell disease or with pre-existing kidney disease were not included.

The present analysis has four major limitations. First, we found no more than 57 individually documented cases of kidney tubular damage caused by deferasirox. Second, the reporting completeness was excellent only in a minority of cases. Third, it has been suggested that the assessment of tubular proteinuria, which consists of low-molecular-weight proteins such as retinol-binding protein or ß_2_-microglobulin and enzymes such as N-acetyl-ß-glucosaminidase, might be an early and accurate marker of tubular dysfunction [37]. Regrettably, we currently have no data to support or infirm this recommendation. Fourth, available data do not allow to estimate the prevalence of the tubular damage unassociated with acute kidney injury caused by deferasirox. Future work is necessary to address these issues.

## 5. Conclusions

Aminoglycoside-class antimicrobials, nucleotide reverse transcriptase inhibitors, and platinum compounds occasionally cause a dose- and time-related kidney disease. Similar damage may be observed on treatment with the iron-chelating agent deferasirox but not with deferoxamine or deferiprone. Deferasirox-associated kidney damage is dose-dependent and more likely to occur when iron stores are low. It has therefore been recommended [4,34] that physicians prescribe the lowest possible dose to achieve a satisfactory iron burden and consider discontinuing therapy if circulating ferritin is 1000 μg/L or less.

## Figures and Tables

**Table 1:** Characteristics of 57 patients 3 to 78, median 15 years of age with kidney damage on deferasirox therapy. Data are presented as median [with interquartile range] or as frequency (with percentage).

Gender		
Female, *N* (%)	27	47
Male, *N* (%)	30	53
**Age**		
Years, median [interquartile range]	15 [6.7–21]	
≤18 years, *N* (%)	35	61
**Underlying transfusion-dependent disease**		
Thalassemia syndrome, *N* (%)	46	81
Diamond Blackfan anemia, *N* (%)	5	8.8
Allogenic stem cell transplantation, *N* (%)	3	5.3
Other conditions ^◆^, *N* (%)	3	5.3
**Deferasirox dose** **^☩^**		
20–30 mg/kg/day, *N* (%)	46	87
31–42 mg/kg/day, *N* (%)	7	13
**Duration of deferasirox therapy ^✙^**		
≤1 month, *N* (%)	5	20
2–6 months, *N* (%)	4	16
>6 months, *N* (%)	16	64
Time to recovery after therapy withdrawal *		
Information not given, *N* (%)	37	65
≤1 week, *N* (%)	2	3.5
2–4 weeks, *N* (%)	3	5.3
>6 months, *N* (%)	5	8.8
Persistent abnormalities reported	2	3.5
Deferasirox therapy rechallenge, *N* (%)	18	32
Relapse of kidney damage, *N* (%)	9	16

^◆^ non-hereditary hemochromatosis, Ewing’s sarcoma, sideroblastic anemia; ^☩^ information not available in 4 cases; ✙ information not available in 32 cases; * deferasirox therapy was not stopped in 17 cases.

**Table 2:** Abnormal urinary findings and electrolyte-acid–base disorders were detected in 57 patients (27 females and 30 males 3 to 78, median 15 years of age) on therapy with oral deferasirox. Data are presented as median and interquartile range or as absolute numbers, as appropriate. Patients with latent tubulopathy, overt tubulopathy without acute kidney injury, and acute kidney injury are presented separately.

	All	Tubulopathy without Kidney Injury		Tubulopathy with Kidney Injury *
		Latent	Overt	
***N***	57	11	37	9
Age, years (median and IQR)	15 [6.7–21]	14 [11–19]	11 [5.6–20]	20 [18–33]
Females/males, *N*	27/30	6/5	21/16	0/9 ^✙^
**Abnormal urinary findings, *N***	54	11	34	9
Renal glucosuria, *N*	34	2	23	9
Tubular proteinuria ^☩^, *N*	21	8	16	2
Excessive total proteinuria, *N*	17	1	11	5
Generalized aminoaciduria, *N*	9	1	4	4
**Electrolyte-acid–base disorders, *N***	46	-	37	9
Metabolic acidosis, *N*	38 ^✿^	-	31	7
Hypophosphatemia, *N*	35	-	27	8
Hypokalemia, *N*	24	-	18	6
Hypouricemia, *N*	11	-	7	4
Hypocalcemia, *N*	6	-	6	0
Hyponatremia, *N*	3	-	1	2

* Stage I in 3, stage II in 5 and stage III in 1 case; ^✿^ anion gap normal in all cases (*N* = 13) with this information; ^✙^ acute kidney injury significantly more common in males than in females (*p* < 0.02); ^☩^ ß_2_-microglobulin excretion in all 21 cases.

## Data Availability

The data supporting this study are available from the corresponding author upon reasonable request.
